# Untargeted Metabolomics for Autism Spectrum Disorders: Current Status and Future Directions

**DOI:** 10.3389/fpsyt.2019.00647

**Published:** 2019-09-10

**Authors:** Kevin E. Glinton, Sarah H. Elsea

**Affiliations:** Department of Molecular and Human Genetics, Baylor College of Medicine, Houston, TX, United States

**Keywords:** autism, untargeted metabolomics, inborn errors of metabolism, metabolome, mass spectrometry, nuclear magnetic resonance spectroscopy

## Abstract

Autism spectrum disorders (ASDs) are a group of neurodevelopment disorders characterized by childhood onset deficits in social communication and interaction. Although the exact etiology of most cases of ASDs is unknown, a portion has been proposed to be associated with various metabolic abnormalities including mitochondrial dysfunction, disorders of cholesterol metabolism, and folate abnormalities. Targeted biochemical testing like plasma amino acid and acylcarnitine profiles have demonstrated limited utility in helping to diagnose and manage such patients. Untargeted metabolomics has emerged, however, as a promising tool in screening for underlying biochemical abnormalities and managing treatment and as a means of investigating possible novel biomarkers for the disorder. Here, we review the principles and methodology behind untargeted metabolomics, recent pilot studies utilizing this technology, and areas in which it may be integrated into the care of children with this disorder in the future.

## Introduction

The term “autism spectrum disorder” (ASD) describes a clinical spectrum of neurodevelopment conditions characterized by deficits in social communication and social interaction with restricted, repetitive patterns of behavior, interests, or activities (1). Although these core features have been recognized for over seven decades (2), only recently have we begun to recognize the largely heterogeneous nature of this disorder, with patients exhibiting varying degrees of impairments, medical complications, and intellectual disability (ID). In the United States, ASDs are estimated to have a prevalence of around 16.8 per 1,000 children older than 8 years (1 in 59), although in some subpopulations, this figure can be as high as 29.3 per 1,000 children (3). Having a child with ASD was found to be associated with an extra $17,081 per year of additional costs to a family, including healthcare-associated costs, adaptive education, therapies, and family-coordinated services (4, 5). Not as easily measured, however, are the associated emotional and social stresses caregivers and family members encounter, often leading to depression, physical complaints, and declines in overall quality of life (6–8).

**Figure 1:**
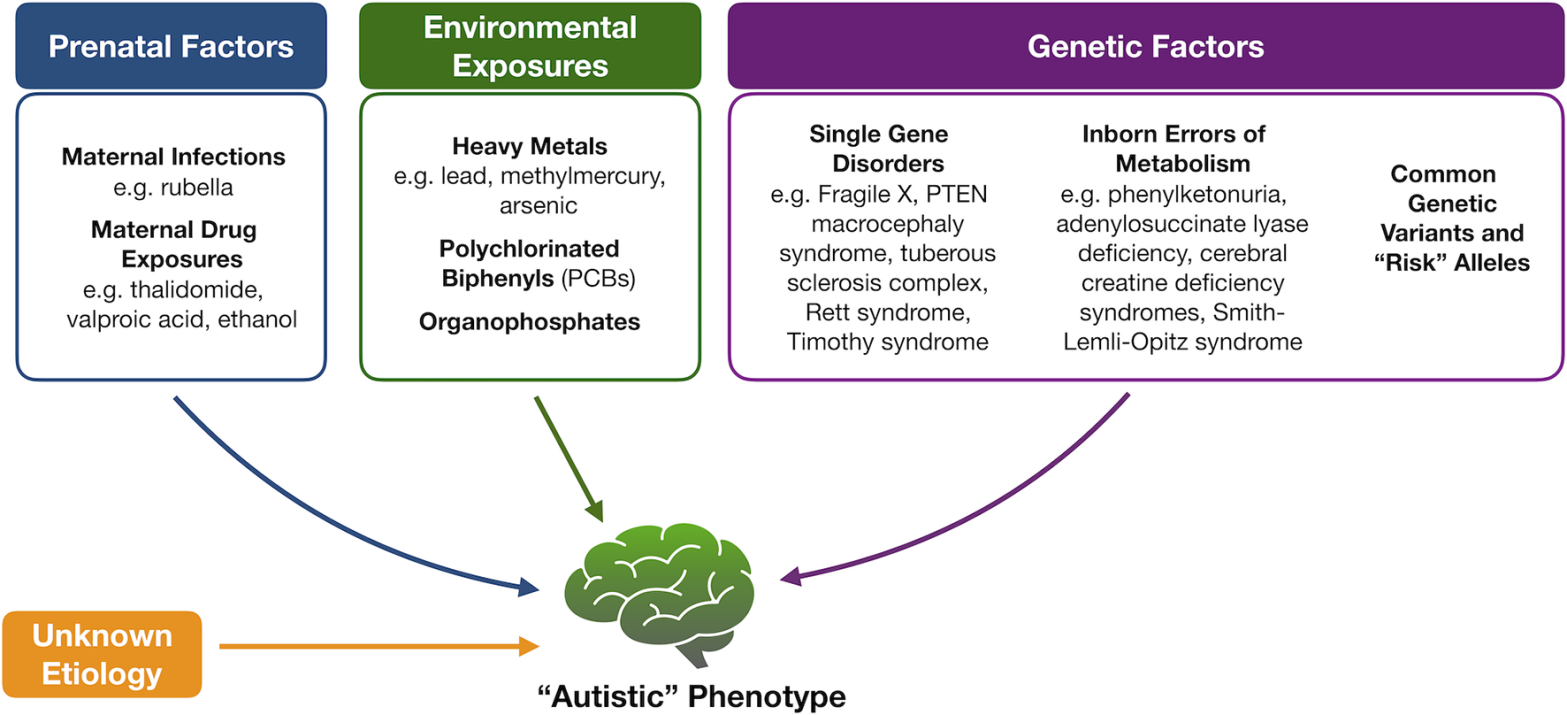
Proposed etiologies for autism spectrum disorders. Multiple factors have been proposed to contribute to the development of autism spectrum disorder, including fetal exposures, childhood exposures, and underlying genetic causes that range from “risk” alleles to known Mendelian disorders and inborn errors of metabolism. In the majority of cases, however, an underlying etiology is not identified.

Because of the heterogeneity associated with ASD, tremendous efforts have been made to identify underlying mechanisms or markers for this complex set of disorders with limited success (**Figure 1**). Genetic variation, for instance, is thought to account for about 50% of the risk for ASD (9). While a portion of cases is thought to be due to common polymorphisms (10, 11), specific molecular diagnoses, like copy number variants or monogenic disorders, are, however, found in an estimated 30% to 40% of children with ASD, prompting the American College of Medical Genetics and Genomics to recommend at least chromosomal microarray and fragile X testing as a first diagnostic step (12). A small portion of ASD cases also occurs in patients with known inborn errors of metabolism (IEMs) (13, 14). The discovery of this association and the potential opportunity for therapeutic intervention have prompted the renewed search for characteristic neurometabolic biomarkers to aid in early screening and diagnosis of patients.

At present, routine metabolic testing has been recommended for patients with autism only on the basis of concerning physical examination features or historical details like seizures, developmental regression, or acidosis, provided the child has passed the relevant state-mandated newborn screening (12, 15). This is in contrast to recommendations from the American Academy of Pediatrics (16) in favor of an initial metabolic workup in patients with ID or global developmental delay (GDD), as these conditions are thought to be more likely of a metabolic nature. This dichotomous approach has raised some concerns including difficulties differentiating (17) the early signs of an isolated ASD from ID/GDD and the paucity of medical providers comfortable with recognizing the signs of an underlying IEM. The identification and use of a specific biomarker would therefore help differentiate these patients and also assist in streamlining the diagnostic process. One of the newest and most promising techniques available for identifying such biomarkers is untargeted metabolomics. Clinical uses of this technology have grown steadily over the past decade; however, its application to the study of autism remains limited for a variety of reasons. Given our current understanding of the various underlying causes, ASD appears to be an ideal disease state in which to apply this technology. Here, we describe the current neurometabolic framework of ASDs, outline the process and scope of untargeted metabolomics, survey the recent applications of untargeted metabolomics in individuals with ASD, and suggest ways in which this technology may expand in the future.

## Metabolomic Profiling—A Primer

**Figure 2:**
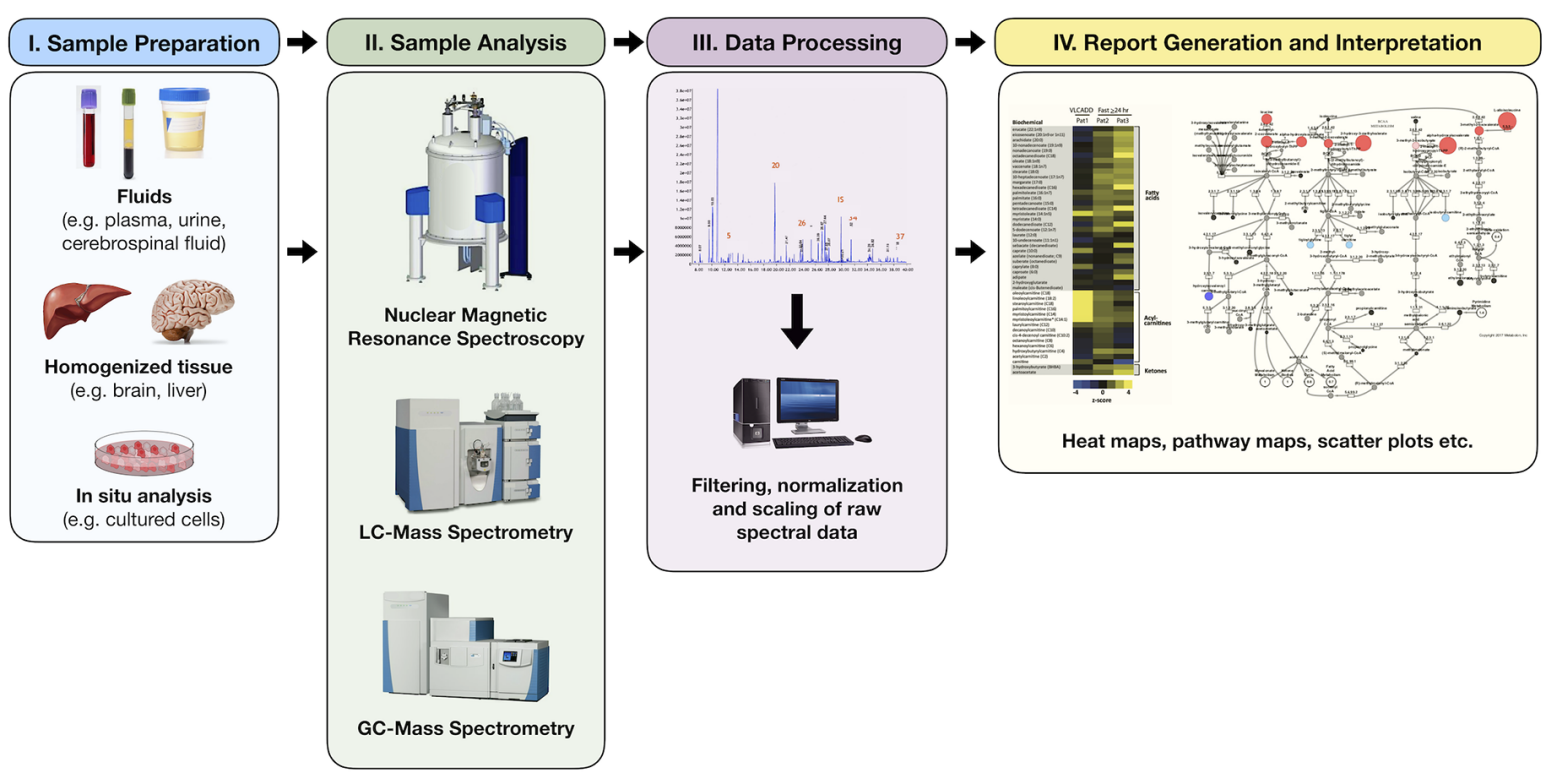
Simplified process of metabolomic profiling. Samples are collected and undergo an initial processing step that may include homogenization and/or centrifugation. This is followed by analysis via nuclear magnetic resonance or mass spectrometry techniques from which raw spectral data are collected. Raw data are then filtered, normalized, and scaled to ensure that analyte peaks may be accurately interpreted. Chemical compound libraries are utilized for accurate compound identification. The resultant data may then be plotted or reported in a variety of ways including heat maps, pathway enrichment maps, and scatter plots.

The study of metabolomics refers to the systematic identification and quantitation of all metabolites in a given organism or biological sample (18). This collection of molecules, known as the *metabolome*, is thought to directly reflect the biochemical activity of the study system at a specific point in time. Metabolomic profiling is not a new concept, however. Archibald Garrod, for instance, first sought to describe the biochemical features of alkaptonuria in urine over a century ago (19). Decades later, Dalgliesh et al. (20), Gates and Sweeley (21), Horning and Horning (22) and Devaux et al. (23) were able to describe the characteristic profiles of various urinary and blood metabolites in the early 1960s–1970s using gas chromatography (GC) methods. In the last decade, however, technologic advancements in chromatographic and analytic techniques have allowed for a tremendous growth in the field of metabolomics, making it possible to identify hundreds, and sometimes thousands, of unique human analytes, leading to unprecedented insight into countless biochemical pathways (**Figure 2**).

Technically, metabolomic data are usually generated by use of nuclear magnetic resonance (NMR) spectroscopy and/or mass spectrometry (MS) (24). Nuclear magnetic resonance spectroscopy uses radiofrequency (RF) waves to generate an electromagnetic field around a biological sample. Variations in this electromagnetic field lead to differential energy absorption and re-emission resulting in a spectrum of emitted RF waves corresponding to each metabolite (25). Although less commonly used for human samples, NMR has the advantage of minimal sample preparation and being able to not only measure metabolite concentration but also identify the chemical structure. Mass spectrometry, on the other hand, makes use of the isolation of gas-phase ions based on their mass-to-charge ratio (m/z) (26). A sample is first separated by use of either a liquid chromatography (LC) or GC column and then ionized (27, 28). Individual peaks are generated for each metabolite with peak intensities corresponding to its relative concentration within the sample. Mass spectrometry–based methods have the advantage of greater sensitivity when compared to NMR, allowing for a larger number of measurable metabolites (29). Mass spectrometry protocols and analytical techniques, however, may vary greatly from one laboratory to another, making standardization and reproducibility of results more difficult when compared to NMR.

Metabolomic studies may be conducted as either *targeted* assays, where only a specific subset of metabolites is measured, or *untargeted*, where as many metabolites as possible are measured without filtering or bias. Both methods have their advantages and disadvantages, with the choice of test usually based upon experimental design and the specific hypothesis being tested (30). Targeted metabolomics, for instance, may be more desirable for the study of a single biochemical pathway where analysis can be focused on only a small group of molecules of interest. This approach has the advantage of limiting the analytical and statistical burden of an experiment, although one risks missing perturbations of secondarily interconnected pathways. Untargeted metabolomics, on the other hand, has the advantage of revealing unique and previously unrecognized changes in metabolites and enzymatic pathways, although the quantity and complexity of experimental data can be challenging (31, 32).

Regardless of the technique, however, it is now theoretically possible to perform metabolomic profiling on almost any tissue, opening up a wealth of potential biochemical information (33). Naturally, metabolomic profiling has found extensive use in the diagnosis and treatment of IEMs, although the test has also helped establish new or previously unrecognized aspects of several other genetic conditions, as well (34–36). In addition, metabolomic profiling has also been used to study to a broader variety of human diseases including obesity and insulin resistance (37, 38), myocardial ischemia and other models of cardiovascular disease (39, 40), hepatocellular disease (41, 42), and malignancies (43, 44).

## Autism and Inborn Errors of Metabolism

Inborn errors of metabolism are themselves rare diagnoses with combined incidences between 1 in 800 and 1 in 2,500 individuals (45–48). It is estimated that only up to 5% (49, 50) of children with ASD will be found to have an IEM, although these disorders are important to consider when confronting a first-time patient. Over the years, many different case reports have attempted to link various IEMs with the development of autism; however, it is important to keep in mind the refinements that have been made over time to the definition of ASD in contrast to other behavioral disorders, developmental delay, and ID. As such, many of these earlier associations have since been shown to be tenuous (51) or at least only single cases, and we therefore restrict our discussion here to disorders in which multiple pieces of evidence support the link between the biochemical findings and the observed clinical phenotype of ASD.

Prior to the advent of universal newborn screening in the United States, many children with these disorders may have initially come to medical attention for “autistic” features and developmental delay. With screening protocols, this is no longer the case, although several IEMs still predispose patients to ASD, despite early identification and adequate treatment (52, 53). We previously demonstrated that untargeted metabolomics may be used to identify many of the diagnostic and even secondary changes in downstream analytes (34). This approach also allows for the complete elucidation of abnormal biochemical pathways and in some cases can offer clues to fundamental abnormalities that may lead to an autistic phenotype in all children.

One example of a known IEM associated with ASD is phenylketonuria (PKU; OMIM 261600), a disorder due to defects in the phenylalanine hydroxylase enzyme system. Children with “classical” PKU have little to no enzyme activity and as a result may have serum phenylalanine concentrations many orders of magnitude greater than unaffected individuals. Metabolomic studies on patients with PKU have demonstrated elevated serum levels of phenylalanine derivatives, including phenylpyruvate, phenylacetate, phenyllactate, mandelic acid, 4-hydroxyphenylacetic acid, and several others (54, 55). It remains unclear which of the abnormal analytes, however, truly lead to the toxic effects observed in patients. Based on urine studies, compounds like phenylpyruvate and phenylacetate have been proposed as more efficient markers for disease than direct measurement of phenylalanine, given ease of obtaining urine samples and a low false-negative diagnostic rate (54). Along with possible direct toxicity, elevated phenylalanine levels are thought to decrease the cerebrospinal fluid (CSF) concentrations of tyrosine and its downstream products like serotonin and dopamine. This hyperphenylalaninemia is also thought to inhibit the transport of large neutral amino acids across the brain, decrease protein synthesis within the brain, and increase myelin turnover with eventual hypomyelination, all processes that may or may not contribute to the development of autism (56, 57).

Without early treatment, PKU leads to severe intellectual impairment along with physical features like fair skin, spastic gait, and microcephaly (58). While the incidence of PKU has remained steady at around 1 in 11,400 live births in the United States (59), newborn screening and the early institution of various treatment modalities have greatly reduced the number of severely affected individuals. Prior to newborn screening, up to 21% (60) of cases of “autism” were later found to have biochemical evidence of untreated PKU, and in a recent study, about 5.7% of untreated patients were diagnosed with ASD (61). Overall, however, in countries where universal newborn screening has rendered “symptomatic” PKU almost nonexistent, it is estimated that only 0.7% (62) of patients with a diagnosis of PKU will meet the criteria for ASD. While this figure is significantly lower than previous estimates, it is still higher than age-matched controls, highlighting a possible link between the biochemical abnormalities in PKU and a susceptibility to ASD.

Autistic features also appear to be a commonly observed in Smith–Lemli–Opitz syndrome (SLOS; OMIM 270400), an autosomal recessive disorder of cholesterol biosynthesis occurring in approximately 1:20,000 to 1:40,000 live births (63–66). Affected individuals are deficient in the enzyme 7-dehydrocholesterol reductase (*DHCR7*; OMIM 602858), the final enzyme in the synthesis of cholesterol, and as a result exhibit abnormally high serum concentrations of 7-dehydrocholesterol and its isomer 8-dehydrocholesterol (67). Total cholesterol levels appear to correlate inversely to the severity of symptoms, although in most patients there is still usually some detectable cholesterol in serum. Cholesterol levels are, however, severely decreased within the brain parenchyma where cholesterol is known to play important roles in embryonic and fetal brain development, myelination, and ongoing synthesis of neurosteroids (68–71). Studies suggest decreased central nervous system (CNS) cholesterol as the cause of the neurodevelopment manifestations of SLOS, as well as the high incidence of ASDs in this population (72). Approximately 50% of children with SLOS also fulfill clinical criteria for a diagnosis of an ASD, suggesting a common pathomechanism between the two disorders (52). Interestingly, this hypothesis is also supported by the finding of significant hypocholesterolemia in up to 20% of children with isolated ASD (73).

The biochemical defect known as adenylosuccinate lyase (ADSL) deficiency (OMIM 103050) has also been described as a disease particularly associated with the development of ASD. Although rare, with an estimated prevalence of only 1 in 1,240,710 individuals (74), approximately 30% (75) of patients with ADSL deficiency exhibit autistic features along with other findings like developmental delay, encephalopathy, seizures, and growth retardation. The disorder is caused by an autosomal recessive defect in *ADSL* (OMIM 608222), which encodes the enzyme ADSL involved in the de novo synthesis of purines. The disease is characterized biochemically by the presence of the compounds succinylamino-imidazole carboxamide riboside (SAICA riboside) and succinyladenosine (S-Ado) in the CSF, plasma, and urine of affected patients (76, 77). Although both compounds are easily assayed through targeted testing, ADSL deficiency is easily identified on untargeted metabolomics by strikingly high levels of S-Ado in both plasma and CSF (78). Accumulated SAICA riboside is thought to be the primary neurotoxicant involved in this disorder, based on animal models (79). Deficiencies in ADSL are also believed to lead to decreased purine levels within the CNS, although the exact physiologic consequences of this are somewhat unclear. There is also some evidence that ADSL deficiency may lead to altered cerebral energy metabolism through the interactions between fumarate and AMP with purines (80).

Finally, much insight into the underlying mechanism of ASD may also be gained from the group of disorders known as cerebral creatine deficiency syndromes (CCDSs). This group of abnormalities comprised the three enzymatic defects in the synthesis of creatine:arginine:glycine amidinotransferase (AGAT) deficiency (OMIM 612718), guanidinoacetate methyltransferase (GAMT) deficiency (OMIM 612736), and SLC6A8 deficiency (OMIM 300352). The creatine:phosphocreatine system plays an essential role in ongoing energy metabolism by serving as a reservoir for phosphate moieties in their transfer back and forth from ATP to ADP (81). In humans, creatine may be either taken in through the diet or synthesized de novo from precursors via the activity of the enzymes AGAT and GAMT in tissues like the kidney pancreas and liver. The molecule may then be transported into its target tissues via the action of its sodium chloride–dependent channel (SLC6A8). Genetic abnormalities in any of these three components lead to a severe deficiency of creatine within the brain parenchyma, with the diagnostic hallmark of the disease being an almost absent creatine peak on ^1^H NMR spectroscopy. While it remains unsettled as to what proportion of creatine within the fetal brain is imported versus synthesis natively, it is clear that this small molecule plays an integral part in ongoing neurodevelopment (82).

Clinically, patients with CCDS exhibit a combination of developmental delay particularly in expressive language, ID, and autistic behaviors (83). The incidence of autistic features appears to vary for each of the three disorders. Approximately 78% to 95% of patients with GAMT deficiency have been reported to have autistic features, while only 41% of patients with SLC6A8 deficiency were felt to exhibit the same behaviors (84–86). As the rarest of the CCDS, accurate estimations of the occurrence of ASD in AGAT deficiency are difficult, although 4 of 16 total patients (25%) were reported to have features of autism in one recent study (87). The phenotype is thought to arise due to deficits in cerebral energy production during and after fetal development, the accumulation of the potentially neurotoxic guanidinoacetate and other guanidino compounds, and possible abnormalities in ongoing neural signaling associated with abnormalities in the creatine–phosphocreatine system (88). Interestingly, treatment of patients with creatine supplementation and arginine restriction in the case of GAMT patients does seem to improve certain aspects of their phenotype, although not all concerns are eliminated. GAMT patients, for instance, do experience improved seizure control and a decrease in movement disorder symptoms, although severe ID remains. Patients with AGAT deficiencies do appear to fare better with some catch-up development and even prevention of symptoms entirely in a prenatally known case. Creatine supplementation does not, however, appear effective for patients with SLC6A8 deficiency, as increased plasma levels are not able to compensate entirely for abnormal transport across the blood-brain barrier.

Other IEMs have been associated with an increased incidence of ASD. These include so-called classical disorders like propionic academia (OMIM 606054) (53), various urea cycle disorders (89), and Sanfilippo syndrome (OMIM 252900, 252920) (90), as well as rarer disorders like succinic semialdehyde dehydrogenase deficiency (OMIM 271980) (91) and branched-chain ketoacid dehydrogenase kinase deficiency (OMIM 614901) (92).

## Autism and Nonspecific Metabolic Disturbances

While known IEMs are informative as to the development of ASD in a subset of patients, there are also other nonspecific metabolic abnormalities that have been explored within this population. Given that the metabolome of different body compartments may consist of hundreds to thousands of compounds, subtle changes identified in a clinical condition can also lead to clues to its mechanism or even treatment. It is important to mention briefly the emerging work (93, 94) on interactions between gut microbiota and the development or even exacerbation of autistic symptoms. It is now well recognized that humans exist in constant symbiosis with thousands of species of microorganisms. Microorganisms have been postulated to help defend against the colonization of pathogenic species and aid in the synthesis of some vitamins and nutrients, as well as help educate the nascent immune system (95–97). Evidence has begun to emerge, however, on the role these microorganisms, particularly in the large intestine, may play in neurodevelopment and behavioral phenotypes with dysbiosis in particular, proposed as a possible mechanism for the development ASD (98–101). Both targeted and untargeted analyses have been used to identify microbiome-associated metabolites in fecal samples (102, 103), as well as effects on plasma/serum (104, 105) lending further evidence to the importance of these organisms. Fecal concentrations of compounds like isopropanol, p-cresol (103), and various short-chain fatty acids (106) have been demonstrated to be altered in patients with ASD. Similarly, human plasma studies in patients with ASD have demonstrated abnormal branched-chain amino acid levels (105), while transfection of mice with microorganisms from these individuals has also led to behavioral changes in mice in multiple studies (104, 107). This particular area of research has grown exponentially in the last decade and will only continue to grow further in the future as assay techniques and methodologies improve.

Mitochondrial dysfunction has been explored as a possible cause or contributor to an autistic phenotype for several decades. Coleman and Blass (108) first identified the presence of lactic acidosis in a small set of patients in 1985, with Lombard (109) later proposing that ASD may be at least partially due to abnormal neuronal oxidative phosphorylation. Since that time, numerous studies have emerged attempting to further delineate the exact role of mitochondria in ASD, with several excellent reviews and meta-analyses on this single topic alone (110, 111). Mitochondrial dysfunction would appear to be the ideal candidate for a possible cause for ASD given the critical role mitochondria play in ATP production, calcium homeostasis, and synaptic plasticity within neurons (112, 113). Mitochondrial ATP production primarily involves the five respiratory complexes and two electron carriers that collectively make up the electron transport chain: NADH dehydrogenase, succinate dehydrogenase, coenzyme Q10 (CoQ10)–cytochrome C reductase, cytochrome C oxidase, ATP synthase, and the electron carriers CoQ10 and cytochrome C. In such a highly regulated and intricate process, it is easy to imagine that any number of molecular changes would have significant effects on overall energy production and neural function.

Mitochondrial dysfunction has been identified in a subset of individuals with ASD utilizing biochemical markers, clinical criteria, direct enzyme assay, and molecular methods. Oliveira and colleagues (114), for example, found that 20.3% of patients in their cohort had elevated plasma lactate levels. Eleven of these patients eventually underwent muscle biopsy, and five of the patients (7.2%) exhibited clearly deficient respiratory chain enzyme activity, most commonly in complexes I, IV, and V. Aside from two patients with suggestive family histories, these patients were otherwise indistinguishable from the remainder of the cohort with no seizures, loss of motor skills, or abnormal physical examinations. This lack of physical findings or suggestive medical histories, however, has been challenged by a similar later study by Weissman et al. (115). That group identified 25 patients with autism and either molecular or enzymatic evidence of a primary mitochondrial disorder, and all of the patients had met clinical criteria for “probably or definite” mitochondrial disease by the Modified Walker Criteria or the Mitochondrial Disease Criteria. In this cohort, patients exhibited a number of systemic complications including seizures (20%), exercise intolerance (68%), gastrointestinal dysfunction (64%), and gross motor delays (32%). Twenty of the patients (80%) had confirmed respiratory chain complex deficiencies on direct enzyme analysis, with 16 patients having complex I defects, in particular. Two patients had likely pathogenic mtDNA point mutations (A3397G, A4295G), while a further four patients had variants of uncertain clinical significance. Other cases of pathogenic mitochondrial variants have appeared sporadically throughout the literature. Graf et al. (116), for instance, described a family affected by the variant G8363A, which encodes the mitochondrial transfer RNA^Lys^. The variant was found in a female child with a clinical diagnosis of Leigh syndrome and in her brother, who was otherwise healthy aside from a diagnosis of autism. In this case, the male patient was found to have a lower level of heteroplasmy than his more severely affected sister hinting at a likely causative role for this variant in the development of his autism. Pons et al. (117) also identified the *MELAS*-associated mtDNA variant, A3243G, in two out of five individuals with ASD. Of the other three patients in the study, one patient had evidence of mitochondrial depletion on muscle biopsy, while the other two had mothers known to carry the A3243G variant. The authors speculated that, given variations in heteroplasmy, these subjects may have had clinically undetectable levels in plasma, but higher levels in the brain. There are also reports of differing levels in mtDNA expression, possible mitochondrial depletion, and deletions/copy number variations in small cohorts (118, 119). Investigations into mitochondrial single-nucleotide polymorphisms as susceptibility loci have also taken place, although these have yielded conflicting results over time (120–123).

Mitochondrial dysfunction, when present, appears to be localized to specific regions in the brains of affected patients. A 2011 study on postmortem brain samples, for instance, demonstrated low electron transport chain (ETC) activity levels in the cerebellum, frontal and temporal cortices of younger subjects (aged 4–10 years) when compared to age-matched controls (124). In this case, complexes III and V activity levels were statistically decreased in the cerebellum, while complex I was decreased in the frontal cortex, and complexes II, III, and V were decreased in the temporal cortex. A 2013 study by the same group confirmed deficient complex I activity in the frontal cortex, although complex V was also significantly affected in these cases (106). Deficient ETC activity was also confirmed in temporal lobe samples by Tang and colleagues (125), although in this case the group found significantly decreased complexes I and IV activities when patients were compared to controls.

Oxidative phosphorylation is just one of many functions of mitochondria thought to be critical to neural development. There has also emerged significant work on the role of oxidative stress on the development of autism. Reactive oxygen species (ROS), generated either endogenously or through exposure to toxic environmental substances, may cause significant cellular damage if not disposed of efficiently through the body’s antioxidant systems. These include enzymes like superoxide dismutase and catalase and nonenzymatic compounds like glutathione (GSH). Some posit that ASD may emerge when the balance between these two systems is somehow upset either through the cumulative buildup of ROS or through genetic predispositions in the form of molecular defects in the antioxidant system. Several studies have, for instance, confirmed the presence of increased oxidative stress in plasma in patients with ASD (126). A 2012 meta-analysis, in fact, demonstrated that on average patients with ASD had a 27% lower plasma concentration of GSH with corresponding 45% higher plasma levels of reduced GSH (GSSG) when compared to health controls (127). There are also studies on the sometimes characteristic patterns of oxidative damage within the brain tissue of autistic patients. These experiments have relied on direct GSH and GSSG measurements, as well as the detection of various “marker” compounds for oxidative stress like 3-nitrotyrosine (3-NT), carboxyethyl pyrrole, and 8-hydroxydeoxyguanosine (8-OH-dG) (128–130). Glutathione levels were found to be 34.2%, and 44.6% decreased in the cerebellum and temporal cortices, respectively, when compared to controls (131, 132). Interestingly, relatively little change was seen in the prefrontal cortex of patients, an observation that was also confirmed in a later direct MRS study (133). Levels of 3-NT and 8-OH-dG were similarly elevated within the cerebellum (129, 130, 133).

Defects in carnitine biosynthesis have been postulated to predispose individuals to nonsyndromic autism (134). Carnitine plays a variety of physiologic roles, but most important is its ability to help shuttle long-chain fatty acids into mitochondria for β-oxidation. In humans, approximately 75% of carnitine is obtained from the diet, with the remainder synthesized endogenously from the amino acids lysine and methionine (135). Carnitine deficiency has been associated with complications like cardiomyopathy and myopathy, and decreased carnitine levels have been previously described in a subset of children with ASD (136). In a 2012 study by Celestino-Soper et al. (137), carnitine biosynthesis defects were linked to an increased risk for ASD among males. Patients were found to carry a deletion in *TMLHE* (OMIM 300777), which encodes 6-*N*-trimethyllysine dioxygenase, the first enzyme in carnitine biosynthesis. The deletion is estimated to occur in approximately 1 in 350 males of European descent; however, it is also almost three times more common in families with more than one male diagnosed with ASD. Interestingly, the affected patients did not have evidence of ongoing systemic carnitine deficiencies, although plasma and urine studies were able to clearly demonstrate abnormal carnitine metabolites. It remains unclear whether or not TMLHE deficiency acts as a risk factor alone, or if there are other as yet unidentified toxic metabolites that build up as a result of this particular enzyme deficiency. There continues to be ongoing interest in this possible contributor for ASD, however, because of the potential for an easy and inexpensive treatment in the form of l-carnitine supplementation.

Finally, there has long been an association observed between ASD and immune dysfunction; however, the exact nature of this relationship remains complex (138–141). Although many of the earlier studies in this area were limited to “patient-reported” symptoms only, more recent studies have relied instead on more careful and systematic review of medical records instead. As a result, a greater evidence base suggests a relationship between the pathogenesis of ASD and prenatal and postnatal immune dysregulation. Atladottir et al. (142), for instance, described increased rates of rheumatoid arthritis and celiac disease in mothers of children with ASD and a higher rate of type 1 diabetes mellitus in both mothers and fathers of children with ASD. Immune dysfunction has also been directly demonstrated in individuals with ASD themselves. Careaga et al. (143) showed, for example, that the peripheral blood mononuclear cells of boys with ASD exhibited characteristic immune responses based on lipopolysaccharide or phytohemagglutinin stimulation. Abnormal neuroinflammatory changes have also been demonstrated, including prominent microglia activation and increased inflammatory cytokine and chemokine production (144–146). While most metabolomic studies are unable to assay compounds as large as cytokines, chemokine, or antibodies, immune responses may lead to measurable alterations in amino acids metabolites, glycolytic, gluconeogenic, oxidative stress, and purine intermediates (147). This complex interplay between immune dysregulation and the development of ASD continues to be an active area of ongoing research, and clearly, we are only now beginning to recognize the extent to which other physiologic processes are affected as well.

## Towards Biomarkers for Autism—The Clinical Application of Metabolomics

The variety of metabolic disturbances identified in patients appears to suggest that ASDs may represent a common endpoint for multiple different abnormal neuronal pathways. The question remains: To what extent can any one test or biomarker adequately assay all of the individual pathways involved in such pathways and how can that information be best used to guide individual risk or even treatment? The answer to this question may, in fact, lie in the use of untargeted metabolomics in patients with ASDs. The idea of broadly surveying the metabolic environment of patients is certainly not new, despite the changes in terminology over the years. Jaeken and Van den Berghe (76), for instance, first performed broad metabolic testing on three children with severe developmental delay and autistic features in 1984. The children all had extensive biochemical testing performed including serum ammonia, pyruvate, lactate, uric acid, arylsulfatase A, phytanic acid and amino acids, urine amino acids, organic acids, and mucopolysaccharides, along with CSF amino acids, sugars, and inorganic phosphate. The subjects were all eventually diagnosed with ADSL deficiency, thus demonstrating early on the kind of utility such expansive analyses can have in this population. While large-scale, untargeted metabolomic studies remain relatively uncommon, there have been small-scale attempts to prove the applicability of this strategy. We highlight in **Table 1** the studies published thus far that overall paint a promising picture for the application of this technology. One caveat that must be considered, however, is that many of these studies did not take into account differences in patients’ diets, medications, and supplement use, which may or may not have been contributors to the findings in several cases.

**Table 1:** Previous studies utilizing untargeted metasbolomics in subjects with autism spectrum disorders.

Study Reference	Study Population	Age Range (y)	Tissue	Study Method	Notable Metabolites	
					Increased	Decreased
Yap et al., (148)	39 children with ASD	3–9	Urine	^1^H NMR	Dimethylamine, taurine, succinic acid, *N*-methylnicotinic acid, *N*-methylnicotinamide, *N*-methyl-2-pyridone-5-carboxamide	Hippurate, phenylacetylglutamine
	28 “nonautistic” siblings					
	34 age-matched controls					
Ming et al., (149)	48 children with ASD	6–14	Urine	UPLC–MS/MS and GC–MS	*trans*-Urocanate, glutaroylcarnitine, 3-methylglutaroylcarnitine	Glycine, serine, threonine, alanine, β-alanine, histidine, taurine, carnosine, uric acid
	53 age-matched controls					
Mavel et al., (150)	30 children with ASD	6–14	Urine	^1^H-^13^C NMR	Glycine, β-alanine, taurine, succinic acid	Creatine, 3-methylhistidine
	28 age-matched controls	6–9				
Emond et al., (151)	26 children with ASD	6–14	Urine	GC–MS	Succinic acid, glycolic acid	Hippurate, phosphate, palmitate, stearate, 3-methyladipate
	24 age-matched controls	6–9				
Noto et al., (160)	21 children with ASD	4–16	Urine	GC–MS	Glycolic acid, homovanillic acid, 3,4-dihydroxybutyric acid, tryptophan	Fructose, 1,2,3-butanetriol, propylene glycol
	21 “nonautistic” siblings	4–17				
Dieme et al., (152)	30 children with ASD	5–12	Urine	^1^H NMR, ^1^H-^13^C HSQC NMR, and LC-HRMS	N-acetylarginine, indoxyl, indoxylsulfate, dihydroxy-1*H*-indole glucuronide I	Methylguanidine, desaminotyrosine, dihydrouracil
	32 age-matched controls	4–13				
Bitar et al., (153)	40 children with ASD	3–15	Urine	^1^H NMR, ^1^H-^13^C NMR, and LC-HRMS	Phosphoserine, glutamic acid, nicotinamide ribotide, trigonelline, 5-amino-imidazole-4-carboxamide, riboflavin, glycerol-3-phosphate, chalice acid,	Threonine, creatine, serine, N-acetylphenylalanine, tyrosine, hydroxybenzoic acid, hydroxyproline, urocanic acid, cysteic acid, 2-hydroxybutyric acid, citric acid, guanine, N-amidino aspartic acid, acetylcarnitine, methyl acetoacetic acid,
	40 age-matched controls	3–15				
Kuwabara et al., (154)	25 individuals with ASD	23–39	Plasma	CE-TOFMS	Arginine, taurine	5-Oxoproline, lactic acid
	28 age-matched controls	24–36				
West et al., (155)	52 children with ASD	6–9	Plasma	LC–MS and GC–MS	Aspartate, serine, DHEA-S, glutaric acid, succinic acid	Citrate, creatinine, isoleucine, hydroxyphenyllactate, glutamate
	30 age-matched controls					
Wang et., al., (156)	173 children with ASD	3–6	Plasma	UPLC/Q-TOF–MS/MS	Decanoylcarnitine, pregnanetriol	Sphingosine-1-phosphate, docosahexaenoic acid, adrenic acid, uric acid
	163 age-matched controls					
Rangel-Huerta et al., (157)	20 children with ASD	2–6	Plasma	UPLC–MS/MS	3-Indoxyl sulfate, 6-hydroxyindole sulfate, tryptophan, 5-bromotryptophan, gamma-glutamylmethionine, methionine, ursodeoxycholate, cortisone, sphingomyelins d182, kynurenine, 1-methylnicotinamide, choline phosphate, 4-methyl-2-oxopetane, decanoylcarnitine	Behenate, sebacate decanedione, arachidate, glutamate, aspartate, orotate, 2-keto-3-deoxyglutamate, galactitol, *N*-acetyl-aspartyl glutamate, fructose
	30 age-matched controls	2–6				
Graham et al., (158)	11 individuals with ASD	4–46	Brain	LC-LTQ Orbitrap MS	*N*-carboxyethyl-γ-aminobutyric acid, 5,6-dihydrouridine, 3-methoxytyramine	N/A
	11 age-matched controls	4–33				
Kurochkin et al., (159)	32 individuals with ASD	2–60	Brain	UPLC–MS/MS	Glutathione disulfide, 5-oxoproline	Glutathione, l-γ-glutamyl-cysteine, l-cysteinyl-glycine,
	40 age-matched controls	0–62				

### Urine Untargeted Metabolomics

Many of the original metabolomic studies were conducted on urine samples, understandably due to its readily availability, particularly in children with an underlying neuropsychiatric disorder. While some of these studies have yielded particularly compelling results, as a whole, clearly consistent and conclusive findings have not been shown. Yap et al. (148) described characteristic perturbations in the urine of 39 children with autism when compared to 28 of their phenotypically normal siblings. Proton NMR analysis was able to identify several dozen compounds and revealed that the urine of subjects contained lower levels of hippurate and phenylacetylglutamine (PAG) with higher levels of dimethylamine (DMA). Nicotinic acid metabolites like *N*-methyl nicotinic acid, *N*-methyl nicotinamide, and *N*-methyl-2-pyridone-5-carboxamide were also increased, as were the small molecules taurine and succinate. The group speculated that the observed metabolic abnormalities may have been due to gut microbiota in the case of hippurate and PAG and abnormal energy metabolism in the case of succinate. Given its role in the tryptophan-tryptophan-serotonin-melatonin pathway, the group also speculated that the abnormalities in nicotinic acid and its metabolites may be a contributor to the disordered sleep pattern observed in autistic patients.

These findings were somewhat limited by the overlap of many of the spectral peaks, which presumably led to a relatively small number of unique compounds that could be identified. To mitigate this, Mavel et al. (150) expanded on the use of NMR by performing two-dimensional studies on urine instead. The technique called heteronuclear single quantum coherence (2D-NMR HSQC) uses both ^1^H NMR and ^13^C NMR to generate a two-dimensional metabolite map with each nucleus corresponding to an axis. This decreases the likelihood of spectral overlap and results in the identification of a larger number of metabolites than standard one-dimensional NMR. Using this, the researchers were able to identify more than 150 metabolites in the urine of 30 children with autism and 28 healthy controls. There were once again increased levels of taurine and succinate observed; however, other molecules like glycine and β-alanine were also elevated when subjects were compared to controls. In contrast to the work by Yap et al (148), there were no statistical differences between the two groups in terms of glutamate, DMA, trimethylamine *N*-oxide, or hippurate.

Gas chromatography–MS studies on urine samples have also been carried out by groups both in the US and Europe in rapid succession between 2012 and 2014. The first of these was a larger study performed on 48 children subjects and 53 age-matched controls by Ming et al. (149). Using a combination of tandem ultrahigh-performance LC/tandem MS (UPLC/MS/MS) and GC/MS, the group identified 82 metabolites that were significantly altered in patients with autism. These included decreased glycine, serine, threonine, alanine, β-alanine, histidine, and taurine, along with elevated *trans*-urocanate, glutaroylcarnitine, and 3-methylglutaroylcarnitine. Interestingly, both of the antioxidants, carnosine and uric acid, were also relatively decreased, while there were once again various metabolites associated with altered gut microbiota identified.

This was followed closely by a 2013 study led by Francois Emond et al. (151) describing increased concentrations of succinate and glycolate with decreased hippurate, phosphate, and several other small molecules. A third successive study from a group out of Italy (160) later also found statistically significant elevations glycolic acid, homovanillic acid, 3,4-dihydroxybutyric acid, and tryptophan. Elevations in glycolate have been associated with primary oxaluria type I; however, the phenomenon was also believed to be associated with yeast overgrowth. Both homovanillic acid and tryptophan, meanwhile, are metabolites of neurotransmitters suggesting once again the integral role of these molecules in psychiatric disorders. Finally, 3,4-dihydroxybutyric acid may be a normal component of human urine, although increased levels have been observed in cases of succinic semialdehyde dehydrogenase deficiency, a disorder in which patients may also present with autistic features (91).

Recently, in an attempt to fully explore the urine metabolome, Dieme et al. (152) detailed an even more expanded approach of combining urine ^1^H NMR, ^1^H ^13^C HSQC NMR, and LC-HRMS analyses. This kind of study presents a particularly significant statistical challenge given the differences in standardization and statistical analysis between each methodology. To address these challenges, the group performed multivariate analysis using two matched cohorts: a primary “training” subset for the initial identification of significant metabolites and a second “independent validation” subset. The two statistical models were then combined to yield a “data fusion block-scaling model” from which several dozen molecules of interest were determined. These included elevated levels of *N*-acetylarginine, indoxyl and indoxyl sulfate with decreased levels of methylguanidine, and several other compounds that remained unidentified over the course of the study. A similar study was carried out by Bitar et al. (153) in a population of children with ASD from the Middle East. The group examined the urine of 40 children with ASD and 40 age-matched controls and utilized a similar “training-validation” model. The group identified perturbations in several compounds previously outlined including tyrosine, 2-hydroxybutyrate, creatine, and glutamate. Interestingly, this study also identified several newly recognized metabolites including trigonelline, cysteic acid, and guanine. Overall, these results once again pointed to abnormalities in amino acid and carbohydrate metabolism, as well as differences in oxidative stress pathways.

### Plasma Untargeted Metabolomic Studies

Metabolomic studies on plasma have thus far tended to roughly corroborate the findings observed in urine, although specific differences have been proposed to be due to differences in renal clearance for some compounds. Kuwabara et al. (154), for instance, found that patients with autism had significantly elevated plasma levels of arginine and taurine with correspondingly low levels of 5-oxoproline and lactic acid. The group utilized a technique known as capillary electrophoresis time-of-flight mass spectroscopy (CE-TOFMS), which relies on the use of an electric field to help separate components before they are subjected to MS. A total of 143 metabolites were identified, and the results of the study were confirmed by absolute metabolite quantification. The abnormalities found once again alluded to the role of oxidative stress and possible mitochondrial dysfunction in patients with ASD.

Significant changes in alternative metabolites were proposed by West et al. (155) a year later following two-tiered analysis of LC–MS/GC–MS data. The group used univariate, multivariate, and machine learning methods to identify significant elevations in aspartate, serine, DHEA-S, glutaric acid, and succinic acid. Decreases in the levels of citrate, creatinine, isoleucine, hydroxyphenyllactate, and glutamate were also found suggesting roles for altered branched-chain amino acid metabolism (isoleucine, hydroxyphenyllactate) and abnormal mitochondrial energy production (succinic acid, DHEA-S, citrate, aspartate, glutamate). Abnormal fatty acid metabolites were the main findings of another study utilizing ultraperformance LC quadrupole time-of-flight MS/MS (UPLC/Q-TOF MS/MS) (156). In this study of 173 total patients with autism, multiple logistic regression models identified 11 total metabolites that could be used to discriminate patients from matched controls. Samples were collected in the fasted state, and patients were instructed to follow a “standardized” diet and exercise regimen. Identified discriminative compounds included sphingosine 1-phosphate (S1P), decanoylcarnitine, pregnanetriol, docosahexaenoic acid (DHA), adrenic acid, and uric acid. The compounds S1P and DHA were felt to be most predictive of autism.

More recently, Rangel‐Huerta et al. (157) published their findings on untargeted metabolomics of plasma samples from 30 children with ASD and 30 age-matched controls. The team subdivided ASD patients into groups consisting of those with some form of neurologic regression (AR) and those without (ANR). Utilizing HPLC–MS/MS, metabolic intermediates in the malate–aspartate shuttle, urea cycle, glucose–alanine cycle and beta-alanine, aspirate, and tryptophan breakdown pathways were found to differ significantly between controls and patients with ASD. Within the two subgroups, however, significant differences were found in the levels of the fatty acids decanoylcarnitine, arachidate, laurate, octanoylcarnitine, and myristate, as well as 7-methylurate and quinate.

### Brain Untargeted Metabolomics

Metabolomic studies on brain tissue and CSF are somewhat lacking with, for instance, no studies thus far on CSF in patients with autism. The examination of postmortem brain samples, unlike studies on other tissues, however, brings with it unique challenges. The brains of patients with ASD have, for instance, been demonstrated to be slightly larger than age-matched controls (161). A few patient samples have also demonstrated thickening of the subependymal cell layer, heterotopias reflecting abnormal neuronal migration and multifocal cerebral and cerebellar dyplasias (162). Further, when conducting metabolomic studies, one has to also bear in mind the biochemical changes that occur in all human brains at the time of death. The human brain is made up of a relatively high proportion of lipids and proteins. The integrity of many of these compounds can be significantly compromised by normal biochemical processes at the time of death, including hypoxic–ischemic changes, inflammation, and apoptosis–necrosis (163, 164). Because of this, it can thus be difficult to determine which metabolomic perturbations are part of the “normal” process of death and which are inherent to ASD. That said, comparison to adequate age- and sex-matched control samples is critical for these analyses.

A study using LC–MS analyses for untargeted metabolomic analysis of brain tissue published by Graham et al. (158) on samples obtained from 11 deceased subjects with autism and 11 controls led to the identification of more than 20,000 “features” (i.e., raw metabolite ion fraction peaks), although further analytic refinements reduced this to 14,328. The group prioritized the isolation of only the most statistically significant features, which resulted in a group of 37 compounds of interest, of which 18 molecules were increased in subjects and 19 decreased. These features were then matched to known spectral signatures so that the true chemical structure of analytes could be definitively identified. The group of statistically significant compounds included *N*-carboxyethyl-γ-aminobutyric acid, 5,6-dihydrouridine, and 3-methoxytyramine. However, when these features were used to build a predictive discriminatory model, none of the resultant statistically significant ion peaks could be definitively identified. While this is unfortunate, the authors did maintain that the study still highlighted the potential of this approach in ongoing biomarker research.

A second pioneering study conducted by Kurochkin et al. (159) examined postmortem prefrontal cortex samples of 32 individuals with ASD and 40 controls using LC–MS. Multidimensional scaling and normalization led to the identification of 1,366 unique analyte peaks with some statistical discrimination incidentally found based on the age of patients. Approximately 15% of the peaks demonstrated significant differences between ASD and control samples. The peaks corresponded with metabolites in several pathways including GSH metabolism, purine metabolism, pyruvate metabolism, propanoate metabolism, TCA cycle, galactose metabolism, starch and sucrose metabolism, nicotinate and nicotinamide metabolism, cysteine and methionine metabolism, and arginine and proline metabolism. The group further investigated how well these metabolite changes correlated with gene expression in different brain regions. Samples from both the frontal cortex and temporal cortex confirmed that genes linked to the altered metabolites were expressed at a higher rate than genes linked to metabolites showing no intensity difference in ASD. Human metabolomic data were also compared with data obtained by analysis of prefrontal cortex samples from two populations of nonhuman primates (chimpanzees and macaques). Once again, the metabolomic changes observed in humans with ASD proved to be unique to affected humans.

## The Future of Metabolomics in Autism

Multiple lines of evidence have suggested over the years that children with ASDs are biochemically different than their peers. Yet it is only now, with the necessary advances in technology, that we have been able to narrow these down to the point where specific markers may one day be found. Indeed, the establishment of analyte databases like ChemSpider (165) and the Human Metabolome DataBase (166), along with integration tools like the Kyoto Encyclopedia of Genes and Genomes (167) and MetaboAnalyst (168), has enabled easier feature annotation and allowed for an expansion of the number of analytes that can be definitively identified.

As with any test or methodology, however, several key issues have been raised as potential impediments to its broader use. First, one has to bear in mind that untargeted metabolomic analyses, in their present form, are still relatively new and in some ways still require a particularly specialized set of skills to adequately interpret. If then used primarily as a screening tool, it would be difficult to educate and train community pediatricians, psychiatrists, and other care providers on the proper interpretation and use of such testing given the breadth of diagnostic abnormalities that can be ascertained. As well, one has to consider the implications of broader “screening” efforts. Certainly, untargeted metabolomics would be useful in ruling out IEMs, but whether or not this kind of testing would be most appropriate incorporated into newborn screening programs versus primarily used for confirmation of a clinical diagnosis remains to be seen (169, 170).

There are also many challenges encountered in the course of investigative experiments using this technology, at seemingly all stages. When planning these types of studies, for instance, one has to consider not only the choice of platform but also the number of both experimental and control samples to be assayed as these can significantly impact both feature identification and statistical analysis. The choice of sampling method also has significant implications as the number of metabolites will differ significantly. Investigators must ensure that all samples (subjects and controls) are collected and processed as uniformly as possible given that most platforms can quite easily reveal signs of improper handling (e.g., hemolysis, anticoagulation additives, improper solvent use, etc.), nutritional supplementation, environmental exposures, and fasting status. While we have detailed the various strengths and weaknesses of each analytical method, each requires careful calibration with appropriate standardization to ensure that results are of high quality. Despite these considerations, experimental errors may still be encountered. Batch effects, for example, may arise when a subset of samples exhibits abnormalities due to differences in retention times, reagents handling, and so on (171). Matrix effects and carryover are also phenomena commonly encountered in MS-based techniques and need to be addressed as well (172). Challenges remain even after analysis is carried out. While feature identification, for instance, has improved significantly, this area can be the most time-consuming aspect of studies (173), given the myriad of compounds found in the human body and the many ways in which these compounds may ionize. Multivariate analyses can be used to ascertain the statistically significant metabolite features, although even the use of these methods is still fraught with sources of error and bias (174).

Most studies have operated under the assumption that children with ASDs are largely biochemically homogenous; however, it is more likely that these individuals exhibit a myriad of biochemical phenotypes similar to the large heterogeneity observed clinically. While it would be ideal, for instance, to have a single or even a small number of “marker” molecules to help definitively distinguish these patients from controls, more than likely the answer may lie in identifying larger pathway perturbations or patterns. While some of the studies we have described here have pointed to broader metabolic disturbances like mitochondrial dysfunction or decreased energy metabolism, these results have not been replicable and have focused on only a small subset of compounds.

We have previously described our approach to untargeted metabolomics, which, in contrast to previous research models of pooled “cases” and “controls,” instead takes an “n of 1” approach that we believe efficiently bridges both clinical and research spheres (34, 36, 175, 176). Here, a single patient’s sample is analyzed by UPLC–MS/MS after which the raw spectral data from 700 to 1,000 unique compounds is compared to a “healthy reference population” to generate a comparative *z* score. The use of this kind of control sample allows for the establishment of reference ranges for each compound and therefore facilitates the identification of any compounds or pathways outside the reference range in a single patient. From a clinical perspective, this allows for a comprehensive metabolic screen and identification of patient-specific global abnormalities. Meanwhile, from a research perspective, the approach allows for the gradual attainment and analysis of experimental samples, standardization in terms of compound identification, and quantification along with a reduced reliance on upfront statistical calculations.

Untargeted metabolomics appears to be a promising research tool and has the potential to make significant discoveries about the underlying features of autism. While we believe our approach to be particularly well suited to this kind of work, there are of course many different ways in which this technology could be better adapted to ASD and improved. We have highlighted here the only metabolomic study of brain tissue in this population, and to the best of our knowledge, there have been no such studies conducted on CSF. Given the primarily neurologic manifestations of autism, this represents an important area of future research, particularly given the ease with which CSF may be obtained compared to postmortem brain tissue and the proven feasibility and reliability of its analysis (175). In addition, urine and plasma studies may be further optimized by more stringent characterization of patients’ diets, supplement intake, and even microbiome. The minimization or even elimination of these kinds of variables can have a significant effect on the interpretation of metabolomic analyses. There is as well a great opportunity for combining both metabolomic and genomic data for an integrated and comprehensive approach to both diagnosis and ongoing management.

In the seven or so decades since Kanner’s first descriptions of autism, we have made many strides in terms of diagnoses and treatment. Frustratingly, however, the answer to the question of “why” it occurs in the first place remains elusive. With the dawning of the so-called “omics” era, however, we have moved closer to just such an answer, and untargeted metabolomic studies may be one of the keys to finally making sense of this challenging disorder.

## Author Contributions

Both authors contributed equally to this work.

## Conflict of Interest Statement

KG and SE are employees of Baylor College of Medicine, which has a partnership with Baylor Genetics and derives revenue from genetic testing.
